# Surgical Shock

**Published:** 1891-03

**Authors:** 


### Surgical Shock.

Dr. Cheever, of Boston, makes valuable suggestions on modern surgical practice, upon the causation, aggravation or mitigation of shock. He believes that modern methods, while reducing pain and hemorrhage, have increased the tediousness of operations, the nausea, the exposure while operating, and the consequent low temperature. Primary shock is diminished and secondary shock is increased : nausea is one of the attending symptoms of shock, and is the most dangerous factor in producing anæthesia. He maintains in regard to anæsthetics that they annul pain at the expense of nausea, and considers it an axiom that anæsthesia does not annul the existing, but only the additional shock which the pain in cutting produces. He insists that the tendency of modern surgery is to unduly prolong operations, patients being frequently one and a half and two hours on the table, the older methods of quick surgery being abandoned.

The following are the chief rules of treatment suggested for the prevention or alleviation of shock:

BEFORE OPERATION:

1. Wait for reaction.
2. Give alcohol in some form a quarter of an hour before anæthesia.
3. Anæsthesia to be as short as possible.
4. Operation to be as rapid as is prudent.
5. Dressing to be as short as possible.
6. The patient never to be chilled.

AFTER OPERATION.

1. Persistent and carefully applied dry heat.
2. Nutrient enemata.
3. Hypodermic injection of brandy or some other diffusible stimulant.
4. Quiet and rest, with the head low.—*Kansas Medical Record.*

